# Longer Sperm Swim More Slowly in the Canary Islands Chiffchaff

**DOI:** 10.3390/cells10061358

**Published:** 2021-05-31

**Authors:** Emily R. A. Cramer, Eduardo Garcia-del-Rey, Lars Erik Johannessen, Terje Laskemoen, Gunnhild Marthinsen, Arild Johnsen, Jan T. Lifjeld

**Affiliations:** 1 Sex and Evolution Research Group, Natural History Museum, University of Oslo, 0318 Oslo, Norway; l.e.johannessen@nhm.uio.no (L.E.J.); terje.laskemoen@gmail.com (T.L.); g.m.marthinsen@nhm.uio.no (G.M.); arild.johnsen@nhm.uio.no (A.J.); j.t.lifjeld@nhm.uio.no (J.T.L.); 2 Macaronesian Institute of Field Ornithology, 38001 Santa Cruz de Tenerife, Canary Islands, Spain; garciadelreyeduardo@gmail.com

**Keywords:** sperm morphology, sperm velocity, sperm motility, Macaronesia, chiffchaff species complex

## Abstract

Sperm swimming performance affects male fertilization success, particularly in species with high sperm competition. Understanding how sperm morphology impacts swimming performance is therefore important. Sperm swimming speed is hypothesized to increase with total sperm length, relative flagellum length (with the flagellum generating forward thrust), and relative midpiece length (as the midpiece contains the mitochondria). We tested these hypotheses and tested for divergence in sperm traits in five island populations of Canary Islands chiffchaff (*Phylloscopus canariensis*). We confirmed incipient mitochondrial DNA differentiation between Gran Canaria and the other islands. Sperm swimming speed correlated negatively with total sperm length, did not correlate with relative flagellum length, and correlated negatively with relative midpiece length (for Gran Canaria only). The proportion of motile cells increased with relative flagellum length on Gran Canaria only. Sperm morphology was similar across islands. We thus add to a growing number of studies on passerine birds that do not support sperm morphology–swimming speed hypotheses. We suggest that the swimming mechanics of passerine sperm are sufficiently different from mammalian sperm that predictions from mammalian hydrodynamic models should no longer be applied for this taxon. While both sperm morphology and sperm swimming speed are likely under selection in passerines, the relationship between them requires further elucidation.

## 1. Introduction

In 1970, Parker [1] introduced the idea of sperm competition, namely, that when multiple males copulate with a female in one reproductive bout, their sperm compete to achieve fertilization. As a response to sperm competition, males invest in sperm and ejaculate phenotypes that improve fertilization success [2,3,4,5]. One such adaptation is faster sperm swimming speed, which increases fertilization success in a range of taxa [6,7,8]. Understanding the physiological basis of sperm swimming speed has thus become an important target for research on species with high sperm competition. Given the recurring biological theme that form and function are linked, a growing number of studies have sought to identify how sperm cell morphology affects swimming speed (e.g., [9,10,11,12,13]).

Early studies focused on the hypothesis that the total length of the sperm cell correlates positively with swimming performance; this hypothesis was proposed in part to explain the observation that species with more sperm competition have longer sperm cells (e.g., [14]). However, hydrodynamic models do not support this hypothesis [15]. Instead, models suggest that swimming performance should be more directly impacted by the relative length of the flagellum (which produces forward thrust in the models by beating back-and-forth or in a helical motion) compared to the head (which is thought to only produce drag, proportional to its surface area, [15]). This hypothesis is based on a large body of literature on hydrodynamic models that recognize that, at the scale and speed of sperm cells, viscosity has a vastly greater effect than inertia, such that a cell almost instantly stops making forward progress when it ceases actively swimming [15,16,17]. Several comparative [18,19] and intraspecific [20,21] studies support the hypothesis that a higher flagellum:head ratio increases swimming speed, although contradictory patterns have also been found [11,22]. 

In addition to hydrodynamic considerations, energy availability is likely to be important for sperm swimming speed and may correlate with sperm morphology [23]. Indeed, rodent species with higher sperm adenosine triphosphate (ATP) content have faster-swimming sperm [24]. In sperm, ATP can be generated via glycolysis (which can potentially occur along the entire length of the flagellum) and/or by oxidative phosphorylation in the mitochondria of the midpiece, with the relative contribution of the two mechanisms being unclear [25]. Assuming that most ATP is generated by oxidative phosphorylation, ATP content and therefore sperm swimming speed are predicted to increase with increasing midpiece volume [23]. For species with long midpieces, such as birds in the infraorder Passerides [26], midpiece length has been used as an indicator of volume, such that a relatively long midpiece is predicted to support faster-swimming sperm cells [27]. In birds, support for the links between midpiece length, ATP content, and sperm swimming speed is limited. The midpiece–speed correlation has been found in some studies [27,28,29] but not others [11,30]. Other studies find no evidence for a positive correlation between midpiece length and ATP content [31,32] or between ATP content and swimming speed [31,32,33]. 

In addition to relationships between sperm morphology and swimming speed, where mechanical and physiological links have been explicitly hypothesized, several studies have investigated the relationship between the proportion of motile cells and sperm morphology [11,21,29]. Although several studies find significant associations [21,29], the basis of the relationship is not clear. The proportion of motile cells can show a different pattern than sperm swimming speed in responding to experimental treatments [34,35] or in correlations with sperm morphology [29]. The proportion of motile cells thus appears to be at least somewhat independent from the swimming speed of the motile cells. However, assuming that the number of motile cells inseminated affects the probability of fertilization, the proportion of motile cells in an ejaculate appears to be an important measure to consider [4,6]. 

Sperm form–function relationships thus remain an area of active research for many taxa, and particularly for passerine birds. Sperm swim differently in passerines compared to other birds and animals, with the flagellum remaining relatively straight and the cell progressing by spinning around its long axis, similar to a drill [36]. In the absence of specific hydrodynamic models of this style of swimming, researchers have thus far relied on predictions from generalized models, with varying results (e.g., [18,22]). Both sperm morphology and swimming speed appear to be under selection in birds [8,37,38,39,40,41,42]. Species where females more frequently copulate with multiple males have faster [22] and longer sperm cells [18,43], with less variation within and among males in total sperm length [44,45,46,47]. Total sperm length varies at least seven-fold across the group (41.5 to 287.6 µm; [18,48]). 

Here, we test the association between sperm form and function in the Canary Islands chiffchaff (*Phylloscopus canariensis*), an endemic species currently found on five of the Canary Islands but extinct from the rest [49]. Specifically, we test whether sperm swimming speed and the proportion of motile cells correlate with total sperm length, the relative length of the flagellum compared to the head, and the relative length of the midpiece compared to total sperm length within each island population, and whether these correlations differ among islands. We a priori chose to test these morphological features’ relation to sperm swimming performance because of their strong representation in the literature and/or the clearly elucidated mechanical or physiological basis for the hypothesized form–function relationship. Further, we test whether sperm morphology differs across islands (as it does in two other Canary Islands endemics: [50,51]), and we confirm the phylogenetic differentiation of the population on Gran Canaria relative to the other islands [52].

## 2. Materials and Methods

### 2.1. Field Procedures

Males (*n* = 114) were caught on the five Canary Islands where the species currently occurs, using mist nets and conspecific playback during the breeding seasons of 2009, 2010, and 2011 (Table S1). The birds were sampled for blood by brachial venipuncture, sampled for sperm by cloacal massage [53], and fitted with uniquely numbered aluminum rings before being released in their territories. Not all samples were obtained from all males (Table S1), and sperm swimming performance was assessed only in March and April of 2010 and 2011. 

For in vitro sperm motility recordings in the field, the ejaculate was immediately diluted in 40–100 μL (depending on the volume of the ejaculate) pre-heated (40 °C) Dulbecco’s Modified Eagle Medium (Advanced D-MEM, Invitrogen, Carlsbad, CA, USA). Within 30 s after ejaculation, 4.9 μL of the diluted ejaculate was pipetted onto a pre-heated standard microscope slide for sperm analysis (20 μm-depth 2-chamber, Leja, Nieuw-Vennep, The Netherlands), mounted on a MiniTherm slide warmer (Hamilton Thorne Biosciences, Beverly MA, USA), and kept at a constant temperature of 40 °C. Sperm motility was then recorded for 30 s using a digital video camera (HDR-HC1E, PAL, Sony, Tokyo, Japan) mounted on an upright microscope (CX41, Olympus, Tokyo, Japan), with a total magnification of 400×. We recorded six independent locations for each slide to increase the number of sperm measured for each male. The remaining sperm sample was fixed in a 5% formaldehyde solution for later analyses of sperm morphology. For eight males in 2010 and 2011, ejaculate quality was insufficient for video analysis. Two males were sampled twice. For the male sampled in 2009 and 2011, we here include sperm only from the 2011 sampling event, when velocity was measured. For the male sampled in 2010 and 2011, we arbitrarily chose to include the data from 2010, though results were similar with the 2011 sample (not shown, but online dataset includes both captures). 

### 2.2. Sperm Analysis

To measure sperm morphology (*n* = 107 males), a droplet (approx. 15 μL) of the sperm–formaldehyde solution was placed on a microscope slide and air dried. Spermatozoa were examined using a digital light microscope (Leica DM6000 B, Leica Microsystems, Heerbrugg, Switzerland) at a magnification of 160× and photographed with a digital camera (DFC420, Leica Microsystems, Switzerland). We measured the length (±0.3 μm) of the head, midpiece, and tail (the midpiece-free end of the flagellum) of 5–23 (mean ± SD 12.0 ± 1.5) intact spermatozoa per male using Leica Application Suite (v. 2.6.0, Leica Microsystems, Switzerland). Flagellum length was calculated as the sum of the midpiece and tail lengths. Total sperm length was calculated as the sum of head and flagellum. Most measurements (*n* = 99 males) were taken by one person (TL), with samples from Tenerife in 2009 (*n* = 8 males) measured by another person. We have previously demonstrated that measurement repeatability of sperm length is very high and that ten spermatozoa per individual are sufficient to accurately estimate individuals’ mean sperm lengths [54]. We calculated the coefficients of variation in total sperm length within males (CV_wm_) and among males (CV_am_) using the equation (SD/mean) × 100 × (1 + 1/(4n)), to correct for small sample sizes [55]. 

To analyze the sperm motility recordings, we used computer-assisted sperm analysis (HTM-CEROS sperm tracker, CEROS version 12, Hamilton Thorne Biosciences, USA) with a frame rate of 50 Hz and 25 frames (i.e., sperm cells were tracked for 0.5 s) for each of the six filming locations (*n* = 73 males). Each analysis location was visually examined, and cell detection parameters were adjusted using the two interactive quality control plots. Cell detection parameters thus varied slightly between recordings; minimum contrast ranged from 50–100, and minimum cell detection size ranged from 6–10 pixels. Motile objects with elongation > 50 were considered to be non-sperm objects and excluded from all analyses. We recorded the average path velocity (VAP), straight line velocity (VSL), and curvilinear velocity (VCL) of individual cells, as well as the proportion of motile sperm for each male. VAP, VSL, and VCL were all highly inter-correlated (*n* = 73 males, all r > 0.98, all *p* < 0.0001) and gave similar results, so we report only the VCL estimate, which gives the actual point-to-point tracked velocity. Spermatozoa with VAP < 30 μm s^−1^ and VSL < than 25 μm s^−1^ were considered static or drifting and counted as non-motile. For analyses of sperm swimming speed, we excluded non-motile sperm, sperm tracked for less than 10 frames, tracks with straightness score (VSL/VAP*100) below 90 or linearity score (VSL/VCL*100) below 60, non-continuous tracks, and tracks for which the maximum frame-to-frame movement exceeded the average frame-to-frame movement by 4 SD for the same track. The latter restrictions successfully deleted occasional tracking errors in the software. For each of 73 males (Table S1), we analyzed VCL from 25 to 419 (mean 174.64 ± 89.5 SD) unique sperm cells in total.

### 2.3. Statistical Analysis on Sperm

To assess the relationship between sperm morphology and swimming speed, we used the VCL data from individual sperm tracks as the response variable and the mean morphological measures of the male as the predictor (total sperm length, flagellum:head ratio, and midpiece length), in a linear mixed model with male identity as a random effect and island as a fixed effect. Note that this approach ignores sperm morphological variation within males (in contrast to the approach of [9]; video image quality was insufficient in this study to use that approach). Preliminary analysis indicated that sperm swimming speed varied among islands and over time, so we centered morphological variables within sampling events (island–year combinations, each lasting less than a week) to remove the effect of these potential confounds [56]. Note that only one person measured sperm morphology within this data subset, so correction for measurer was not necessary. Initially, island was included as an interaction with each morphological feature, to test whether form–function relationships differed across islands. These interaction terms were removed if they were not significant (*p* > 0.05, [57]). Multicollinearity was not problematic in the final model, as the variance inflation factor was <3 for main effects not involved in interaction terms [57]. Results were highly similar if we instead constructed separate models for each of the morphological variables (using the ratio of the midpiece:total sperm length rather than midpiece length controlling for total length as a covariate) or if we used mean values rather than data from individual cells (data not shown). Significance was assessed using Sattherthwaite’s degrees of freedom in lmerTest [58,59]. 

For the proportion of motile cells, we constructed a generalized linear mixed model with a binomial error structure. The three morphological variables, sperm swimming speed (centered within sampling events), and island were fixed effects; male identity was a random effect; and the number of motile cells compared to immotile cells was the response variable (i.e., equivalent to the proportion of motile cells, combined with the cbind() function). A model including all pairwise interactions between island and other predictors did not converge, so we instead used a forward selection procedure, using likelihood ratio tests to compare a simple model (with main effects and previously-found significant pairwise interactions) to more complex models (including additional pairwise interactions between island and other predictors). Note that lmerTest does not analyze output from generalized linear mixed models, and lme4 does not estimate degrees of freedom, so a test across all levels of island does not return a *p*-value in this framework; a likelihood ratio test comparison of models with and without the main effect of island is not feasible, since island was involved in a significant interaction. We also assessed a simpler model structure using log-transformed proportion of motile cells as the response variable, with all pairwise interactions as predictors in the initial model, followed by backwards removal of interactions. Here, we weighted observations by the total number of cells in the recording. 

To compare sperm morphology across islands, we constructed a separate mixed model for each morphological variable, with male identity and measurer as random effects, and data on individual cells as the response variable. We compared within-male variation in total sperm length across islands in a linear mixed model using the standard deviation in total sperm length as the response variable, island as the predictor, total sperm length as a covariate, and measurer as a random effect [60]; this approach avoids the complications of interpreting ratios [61]. The relationship between sperm swimming speed and relative midpiece length differed for one island compared to the others (see results). We therefore tested whether the correlation between midpiece length and total sperm length differed across islands. To distinguish between-individual and within-individual relationships, we centered the predictor variable for each individual male [62]. The model included a random effect of male identity and fixed effects of the male’s mean total sperm length, the cell’s deviation in total sperm length compared to that male’s mean, and island. We initially included an interaction term of island with each of these morphological predictors, and we removed the interaction if it was not significant (*p* > 0.05). To simplify this model, we included only the 99 males with sperm measured by TL. Finally, we compared the among-male variation in sperm total length across islands using a Levene’s test, using the average measurement per male rather than measures of individual cells. As an alternate approach using data on individual cells, we tested whether allowing each island to have different variance in total sperm length (using the varIdent function) showed improved model fit compared to one where all islands were constrained to have the same variance, using nlme [63]. Results were similar (not shown). All statistics were performed in R (Vienna, Austria).

Sperm measures vary across the breeding season in some species [64,65,66,67,68] but not all (e.g., [53]). However, our sampling scheme did not allow us to assess or control for such effects here. That is, islands were sampled in brief, non-overlapping visits, each lasting less than one week; these visits were unevenly distributed among islands and years. Furthermore, the timing of breeding in the Canary Islands varies depending on elevation and climate [49], such that including day of the year as a covariate would not effectively capture breeding season trends across our field sites. Because our primary interest is the form–function relationship within each island, with data collected over short time spans per island, date effects are unlikely to affect our results. 

### 2.4. Genetic Analyses

We sequenced the mitochondrial *cytochrome c oxidase subunit I* (COI) gene for 49 males (Table S1). DNA was extracted using a commercial spin column kit (E.Z.N.A. DNA Kit; Omega Bio-Tek, Norcross, GA, USA), following the manufacturers’ protocol. The first part (655 bp) of the COI gene was amplified in PCR reaction volumes of 12.5 μL, containing dH_2_O, 1× PCR buffer, 2.5 mM magnesium, 0.05 mM dNTP, 0.1 μM forward and reverse primer, 0.3 U Platinum *Taq* DNA polymerase (ThermoFisher Scientific, Waltham, MA, USA), and approximately 50 ng DNA template. Amplification conditions were 94 °C for 2 min, 35 cycles of 94 °C for 30 s, 55 °C for 30 s, and 72 °C for 45 s, followed by a final elongation step of 72 °C for 10 min. In order to confirm amplification success, 2 μL of the PCR product was electrophoresed in 1% agarose. The remaining PCR product was purified by ethanol precipitation and sequenced using BigDye Terminator v 3.1 Cycle Sequencing kit (Applied Biosystems, Waltham, MA, USA). The sequencing products were analyzed on an ABI Prism 3100 Genetic Analyzer (Applied Biosystems). Sequences were manually edited in CodonCode Aligner 3.7.1. (CodonCode Corporation, Centerville, MA, USA) and aligned using ClustalW. All sequences are available in the dataset DS-NOCIC at the Barcode of Life Database (BOLD) website (www.boldsystems.org, accessed 25 March 2021), with associated sample and primer information, and have been uploaded to GenBank. Translation from nucleotide to amino acid sequences of the analyzed regions in Mega v5.10 revealed no stop codons or frame shift mutations, and there were no systematic double peaks in the COI region, indicating an absence of pseudogenes. We created a minimum spanning network with all unique haplotypes using the package of Pegas [69] based on our COI sequences, as well as performing a combined analysis including sequences from [52].

## 3. Results

### 3.1. Sperm Morphology–Function Relationships

**Figure 1:**
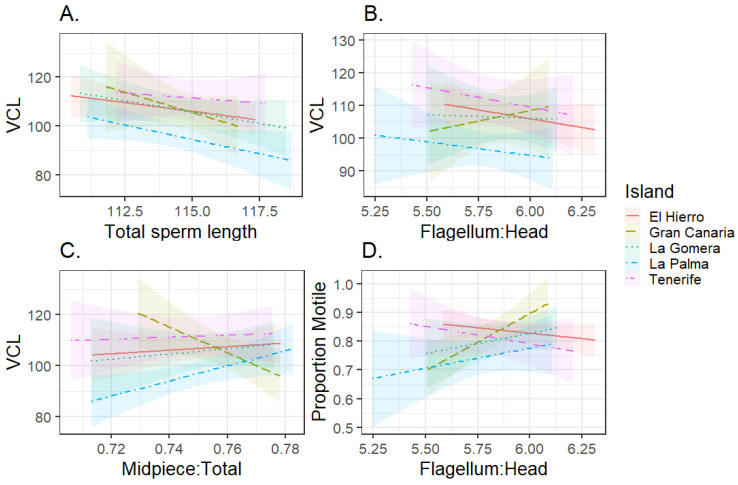
Relationship between sperm morphology and swimming performance for Canary Islands chiffchaffs. (**A**) Total sperm length (µm) versus sperm swimming speed, (**B**) flagellum:head ratio versus sperm swimming speed, (**C**) midpiece:total sperm length ratio versus swimming speed, (**D**) flagellum:head ratio versus the proportion of motile cells. Average values per male are shown, though statistics used curvilinear velocity (VCL, µm/s) and motility data from individual cells. Islands of sampling: El Hierro: solid line, *n* = 21 males. Gran Canaria: dashed line, *n* = 13 males. La Gomera: dotted, *n* = 9 males. La Palma: dot, single dash, *n* = 22 males. Tenerife: dot, several dashes, *n* = 8 males.

Ejaculates with relatively long sperm showed lower swimming speeds in the Canary Islands chiffchaff (Figure 1A, F_1,60.92_ = 8.83, *p* = 0.004, estimated effect ± SE, −1.88 ± 0.63), and this relationship did not differ significantly among islands (interaction term, F_4,57.46_ = 0.41, *p* = 0.80, removed from the model). The mean flagellum:head ratio was not significantly related to swimming speed (Figure 1B, F_1,61.90_ = 1.24, *p* = 0.27; interaction term not significant, F_4,53.50_ = 0.35, *p* = 0.84, removed from model). The relationship between sperm swimming speed and the mean relative length of the midpiece differed significantly across islands (Figure 1C, interaction term F_4,62.04_ = 3.61, *p* = 0.01). Ejaculates with relatively long midpiece, controlling for total length as a covariate, showed lower swimming speed in the Gran Canaria sample (−3.87 ± 1.27, t = −3.05, *p* = 0.003) and higher speed on La Palma (2.44 ± 1.11, t = 2.19, *p* = 0.03), while the relationship was not significantly different from zero for other islands (|t| < 0.41, *p* > 0.68). 

The proportion of motile cells did not relate to total sperm length (z = 0.02, *p* = 0.98), midpiece length (z = 1.63, *p* = 0.10), or VCL (z = 0.09, *p* = 0.93). The relationship between proportion of motile cells and the flagellum:head ratio differed across islands (likelihood ratio test for interaction term, $\chi_{4}^{2}$ = 14.35, *p* = 0.006, Figure 1D). This relationship was significantly positive for Gran Canaria (2.72 ± 0.83, Z = 3.27, *p* = 0.001) and not significant for other islands (estimates between −1.05 and 0.44, *p* > 0.07). In the simpler, fixed-effects model using log-transformed proportion of motile cells, the interaction between island and flagellum:head ratio approached significance (F_4,39.47_ = 2.44, *p* = 0.052).

### 3.2. Among-Island Comparison of Sperm Phenotype and Genetic Divergence

Mean sperm morphology did not significantly differ among islands (F < 1.5, *p* > 0.2, Table 1), nor did within- and among-male variation in total sperm length (F < 1.9, *p* > 0.1, Table 1). Across all individuals, the CV_am_ was 1.87. 

Sperm midpiece length was significantly positively correlated with total sperm length among individuals (F_1,92.92_ = 20.12, *p* < 0.001, 0.47 ± 0.11) and within ejaculates (F_1,1109.86_ = 228.43, *p* < 0.001, 0.55 ± 0.04; note that each individual contributed only a single ejaculate). These relationships did not differ across islands (interaction terms F < 1.9, *p* > 0.12, removed from model). 

Swimming speed differed across islands (F_4,61.60_ = 6.57, *p* < 0.001, in models also including morphological covariates). After controlling for other effects, swimming speed was low on La Palma (pairwise comparisons to all other islands: t > 2.7, *p* < 0.009), with comparisons among other islands being non-significant (|t| < 1.2, *p* > 0.25; Table 1). In the mixed effects model, the proportion of motile cells was lower on La Palma than on El Hierro or Gran Canaria (z > 2.5, *p* < 0.01), but was not significantly different for other comparisons among islands (z < 1.65, *p* > 0.10; Table 1; overall island effect F = 2.99, *p*-value not available due to limitations in estimating degrees of freedom in package lme4). In the simpler fixed effects model, the overall effect of island on the proportion of motile cells was not significant (F_4,34.77_ = 2.15, *p* = 0.09).

**Figure 2:**
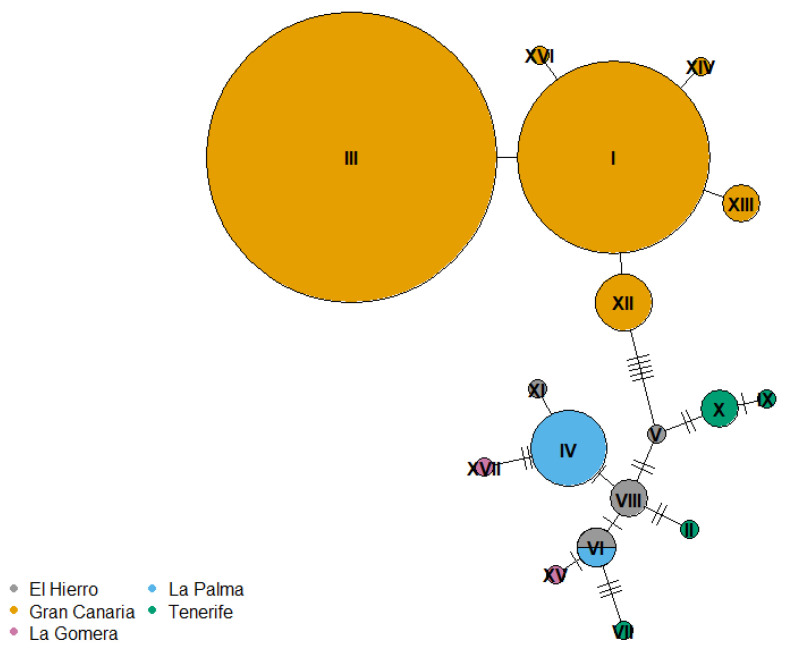
Minimum spanning network of 22 COI haplotypes from 49 individual Canary Islands chiffchaffs sampled in this study, with color indicating the island where the individual was captured. The sizes of the circles are proportional to the haplotype frequencies (smallest circle = 1 individual). Numbers of mutational steps are indicated along the lines connecting haplotypes.

Within the birds sampled in this study, the Gran Canaria population shared no mitochondrial haplotypes with other islands (Figure 2). However, two birds sampled by Illera et al. [52] on Tenerife shared their haplotype with three birds we sampled on Gran Canaria (Tables S2 and S3, Figure S1). Among the other islands, our haplotypes showed high similarity (Figure 2), and haplotypes were intermixed among islands in the combined dataset with Illera et al.’s [52] (Figure S1).

## 4. Discussion

### 4.1. Sperm Morphology–Motility Relationships in Passerines

Results from the Canary Islands chiffchaff do not support predicted form–function relationships derived from fluid dynamic models of sperm swimming. In the Canary Islands chiffchaff, ejaculates with longer sperm showed slower swimming speeds, and the mean flagellum:head ratio was not significantly related to swimming speed. If total sperm length or the flagellum:head ratio directly influenced sperm swimming speed through hydrodynamic effects, these morphological variables should be consistently correlated with swimming speed across species and studies (as also argued by [12]). Contradicting this expectation, correlations can vary substantially among species, with total sperm length being significantly negatively (this study, [70]) or significantly positively [71] correlated with swimming speed, or uncorrelated [13]. Similarly, the flagellum:head ratio can be significantly negatively [11] or positively [32] related to swimming speed, or show no significant relationship (e.g., this study). Correlations can also differ between populations of the same species [11,21], within the same populations before and after a social manipulation [71], and among individuals of the same population with different social dominance status [12]. Comparative studies across species also give varying results depending on the dataset, with some studies finding the predicted relationships [18] and many others finding no significant relationships between these three morphological measures and swimming speed [22,33,72]. Differences in protocols for collecting and measuring sperm morphology and swimming speed can complicate the comparison of results across studies [73]. However, protocol differences are not sufficient to explain the variation we see here, since several studies use the same protocol on different populations or at different time points and observe different results [12,71]. Moreover, individuals whose sperm swims relatively quickly in one experimental medium tend to also swim quickly in other media [34,74], suggesting that a morphology–speed relationship present in one medium might also be expected in the other(s), as seen in [11]. Although additional insights may be obtained by correlating the speed and morphology of individual sperm cells [9], overall, empirical studies in passerines do not provide support for sperm morphology–motility relationships predicted from hydrodynamic models of mammalian-like sperm [15,17].

In addition to this lack of empirical support, passerine sperm swimming mechanics appear to violate the assumptions of those hydrodynamic models. Passerine sperm swims by spinning around its long axis like a drill [36], while hydrodynamic models assume that the flagellum bends, whipping back and forth or around, to create thrust [15,17]. A fundamental difference in the hydrodynamics of a spiraling cell and a non-spiraling cell is suggested by experimental work in bacteria, which have used mechanical [75] or genetic [76] engineering to create spiral-shaped and non-spiral-shaped bacteria, finding that swimming speed differs depending on shape. Hydrodynamic modelling of a crescent-shaped bacteria even suggests that such a cell body can contribute to forward thrust [77], contrasting to hydrodynamic models of sperm where the sperm head (equivalent to the bacterial cell body) only causes drag [17]. Indeed, in passerines, a membrane projects from and wraps helically around the sperm head, and may help the cell to establish the spin by which it moves [72]. Sperm swimming speed increases with the width of this membrane (as well as with acrosome length and width) to intermediate values, while slowing with more extreme values [72]. The combination of a fundamentally different mechanism of swimming, and the absence of clear empirical support for previous models of sperm swimming in passerines, indicates that these hypotheses should no longer be applied to passerines.

### 4.2. Sperm Morphology–Motility Relationships in the Canary Islands Chiffchaff

Mean total sperm length was negatively associated with swimming speed on all of the Canary Islands. Because of the inconsistency in observed morphology–swimming speed correlations across passerine species, this association appears more likely to be explained by an unmeasured, correlated variable than by a direct, hydrodynamic linkage between form and function. The unmeasured correlate could, for example, be a component of the seminal fluid; variation in seminal fluid is thought to explain the rapid changes in sperm mobility in response to the social environment in chickens [78], highlighting the potential for non-sperm components of the ejaculate to modify the swimming performance of the cells. Such variation in seminal fluid may also explain the relationships between sperm morphology and the proportion of motile cells observed for Gran Canaria in this study and in previous studies [21,29]. Further work is required to identify the covariate that underlies the relationship between total sperm length and swimming velocity in the Canary Islands chiffchaff, and between morphology and the proportion of motile cells more generally. 

Two sperm form–function relationships differed on Gran Canaria relative to other islands, which is of particular interest since Gran Canaria is genetically differentiated from the other islands ([52] and this study). Specifically, the relationship between the proportion of motile cells and the flagellum:head ratio was significantly positive, and the relationship between sperm swimming speed and mean relative midpiece length was significantly negative, only on Gran Canaria. Midpiece length is hypothesized to affect sperm swimming speed via an impact on ATP availability [23,27]. However, ATP content is not consistently positively associated with midpiece length and sperm swimming speed across studies [31,32,33]. Variation in midpiece thickness, in the density of mitochondrial cristae, and in efficiency of ATP transport from the site of production to its location of use all may contribute to inconsistencies in the relationship between midpiece length and swimming speed [25,79]. While these possibilities were not assessed, we found no evidence at a gross morphological scale that the relationship between total sperm length and midpiece length differed for Gran Canaria. Our results suggest that selective pressure on sperm may differ on this island, consistent with this population being in an early stage of differentiation from the other islands [52]; it is also plausible that mitochondrial function differs on this island, affecting sperm midpiece performance.

### 4.3. Sperm Evolution in the Canary Islands Chiffchaff

Sperm morphology was relatively similar across all islands, with total sperm length being at most only about 1% divergent between islands. The degree of intraspecific divergence in total sperm length varies substantially among species with, for example, the bluethroat *Luscinia svecica* showing 11.6% divergence among subspecies, and several species showing about 3% intraspecific divergence ([80] and references therein). Sperm morphology is expected to evolve more quickly in species with higher levels of multiple mating by females [43], and the Canary Islands chiffchaff likely has substantial levels of multiple mating. That is, among-male variation in total sperm length can be used to predict extra-pair paternity rates, since it correlates negatively with extra-pair paternity [46,81]. Using the calculator provided by [44], the predicted level of extra-pair paternity in Canary Islands chiffchaffs is 25%; this value is similar to the 23–33% of offspring sired by extra-pair males in the most closely related species with paternity data to our knowledge (*Phylloscopus trochilus*; [82,83]). Selective pressure on sperm due to sperm competition may therefore be substantial in this species, although the timescale for among-island divergence may be limited. Though chiffchaffs are estimated to have colonized the Canary Islands about 2.28 million years ago [84], with genetic divergence from *P. sindianus* and *P. collybita* estimated at about 0.5 Ma [85], the signature of genetic differentiation among Canary Islands chiffchaffs is more recent (estimated at about 29,900 years [52]). 

Alternatively, evolution of sperm morphology may be constrained in the Canary Islands chiffchaff. Though the cause of the negative relationship between total sperm length and swimming speed is unclear, there appears to be a tradeoff between these two variables, as is frequently hypothesized among traits affecting fertilization success under sperm competition (also including, e.g., sperm size, number of sperm ejaculated, and sperm longevity; [4,6]). In passerines, total sperm length and swimming speed may independently increase fertilization success (e.g., potentially affecting compatibility with the female’s sperm storage tubules [86,87,88] and relating to the ability to cross the hostile vaginal environment [37,38]). Producing longer sperm and producing faster-swimming sperm may both be relatively energetically costly, thus causing males to trade off investment into one trait against investment into the other [4,6]. Note that other patterns may also be possible depending on among-male variation in resource availability and allocation to reproduction (e.g., [89,90]) and allocation to sperm competition vs. other reproductive traits (e.g., [91,92]). If males cannot simultaneously increase both sperm swimming speed and total sperm length, there may be antagonistic selection on these traits that helps explain the lack of differentiation in total sperm length across the Canary Islands chiffchaffs. If this relationship extends beyond this species, it may also help explain why total sperm length is currently relatively static throughout the Phylloscopidae [93] and why sperm morphological differentiation between subspecies was not observed in *P. trochilus* [94]. 

## 5. Conclusions

Results from the Canary Islands chiffchaff do not support the most common hypotheses linking sperm morphology to swimming speed, and, indeed, overall support for these hypotheses in passerine birds is weak. These hypotheses were generated from hydrodynamic models of a fundamentally different mechanism of swimming. While they represented a reasonable starting point in the absence of models directly relevant to passerine sperm swimming mechanics, the accumulated evidence strongly suggests a need to move beyond these coarse morphological measures in order to understand how passerine sperm morphology affects swimming speed. In addition, differentiation in the relationships between sperm form and function on Gran Canaria relative to other islands corroborates the genetic differentiation of this population and raises the possibility of a different selective regime for the sperm of Canary Islands chiffchaffs on this island. Both sperm morphology and sperm swimming performance are likely to be under selection due to sperm competition in passerines, but the link between them is not yet well understood.

## Figures and Tables

**Table 1:** Mean ± SD values and statistical comparisons for sperm morphological and motility measurements for the five Canary Islands.

	Island (*n* Males Morphology, Motility)					ANOVA or Levene’s Test
Sperm Trait	El Hierro (23, 21)	Gran Canaria (30, 13)	La Gomera (11, 9)	La Palma (22, 22)	Tenerife (19, 8)	
Total sperm length (µm)	114.19 ± 2.10	114.52 ± 1.55	114.85 ± 2.21	114.19 ± 2.03	114.10 ± 2.92	F_4,101.75_ = 0.33, *p* = 0.86
F:H	5.90 ± 0.21	5.89 ± 0.18	5.82 ± 0.27	5.80 ± 0.21	5.66 ± 0.35	F_4,100.25_ = 0.98, *p* = 0.42
Midpiece length (µm)	85.59 ± 2.73	87.07 ± 1.96	86.40 ± 2.43	85.40 ± 1.85	83.61 ± 6.20	F_4,99.04_ = 1.42, *p* = 0.23
CV_wm_	1.46 ± 0.66	1.52 ± 0.63	1.39 ± 0.78	1.44 ± 0.34	1.37 ± 0.44	F_4,97.15_ = 0.83, *p* = 0.51
CV_am_	1.86	1.37	1.97	1.8	2.59	F_4,102_ = 1.81, *p* = 0.13
VCL (µm/s)	106.77 ± 8.50	105.99 ± 12.50	106.22 ± 7.85	96.41 ± 11.59	111.44 ± 8.07	F_4,61.57_ = 7.49, *p* < 0.001
Proportion motile	0.83 ± 0.06	0.82 ± 0.10	0.81 ± 0.07	0.75 ± 0.13	0.81 ± 0.07	F_4, 34.77_ = 2.15, *p* = 0.09

F:H, ratio of the length of the flagellum to the head. CV, coefficient of variation in total sperm length within (CV_wm_) or among males (CV_am_). VCL, sperm swimming speed, curvilinear velocity. Values here are the averages across the mean per male, while most statistics were performed on cell-level data controlling for male identity. The comparison of CV_wm_ used standard deviation as the response variable, controlling for mean total sperm length as a covariate. Statistical comparisons across islands for VCL and proportion motile are from models that also included morphological covariates.

## Data Availability

Sperm and genetic samples were accessioned to the Natural History Museum (accession numbers in Table S1). COI sequences are available at http://www.boldsystems.org/index.php/Public_SearchTerms?query=DS-NOCIC (accessed date 25 March 2021) and on GenBank (accession numbers MW845010-MW845058). Data on sperm morphology and motility are available in Tables S4 and S5.

## Supplementary Materials

The following are available online at https://www.mdpi.com/article/10.3390/cells10061358/s1, Figure S1: Minimum spanning network of Canary Islands chiffchaffs COI sequences, combining this study with data from Illera et al. [52], Table S1: Overview of Canary Islands chiffchaff samples, Table S2: Assignment of individuals to haplogroups, Table S3: Summary of haplogroup frequencies among islands, Table S4: Sperm morphology data, Table S5: Sperm motility data.
