# Supplementary material for: Longer Sperm Swim More Slowly in the Canary Islands Chiffchaff
Source: Cells. 2021 May 31;10(6):1358. doi: 10.3390/cells10061358 (PMC8228216; doi:10.3390/cells10061358)
Supplement: Supplementary file 1 [file cells-10-01358-s001.zip › cells-1206148-supplementary/Figure S1.pdf]

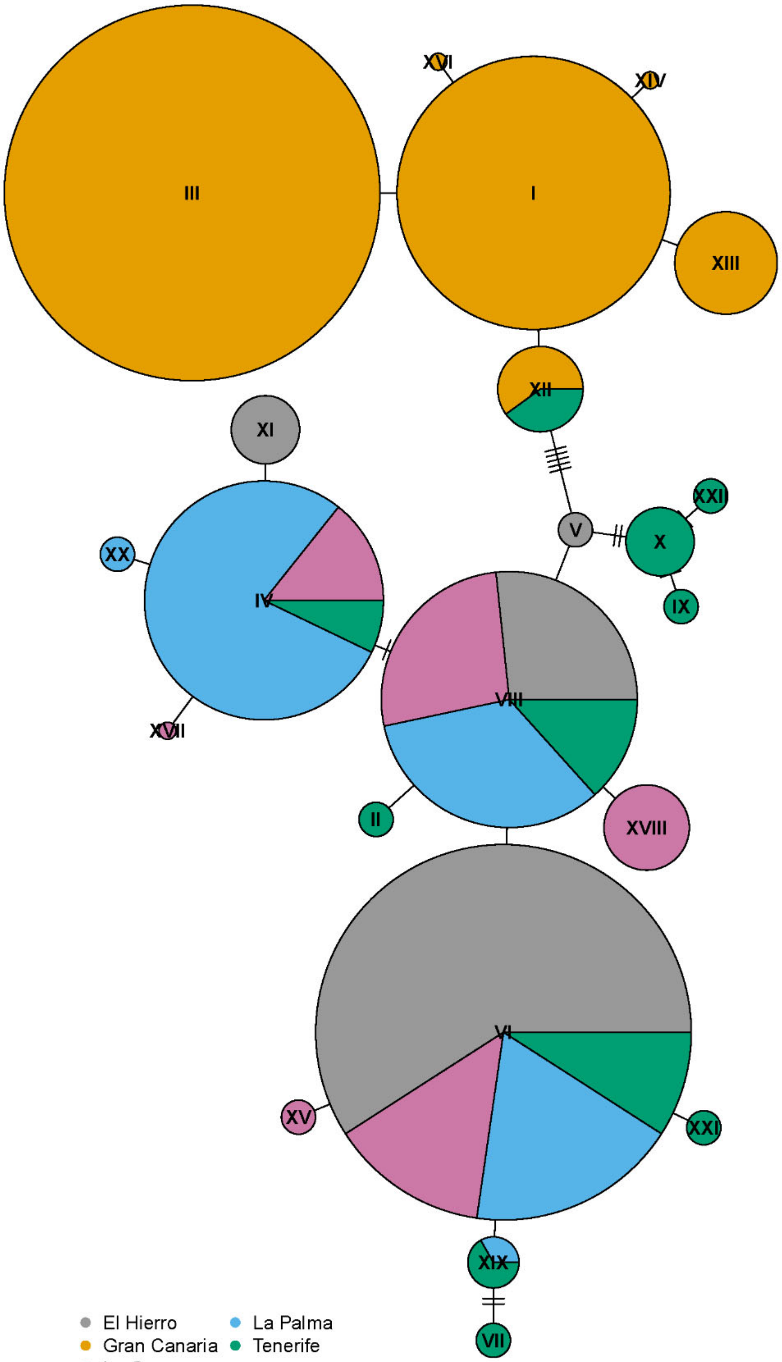

Figure S1: Minimum spanning network of Canary Islands chiffchaffs COI sequences, combining this study with data from Illera et al. [51]. Color indicates the island where the individual was captured. The sizes of the circles are proportional to the haplotype frequencies (smallest circle = 1 individual; count data are in Table S3). Numbers of mutational steps are indicated along the lines connecting haplotypes. Haplogroup numbering corresponds to numbering in tables S1-S3.
